# The Potential Role of Model for End-Stage Liver Disease (MELD)-Sodium Score in Predicting the Severity of Acute Pancreatitis

**DOI:** 10.7759/cureus.33198

**Published:** 2022-12-31

**Authors:** Ali Emre Nayci, Yiğit Düzköylü, Cihad Tatar, Ufuk Oğuz Idiz, Mahmut Kaan Demircioğlu, Mahmut Emin Çiçek, Ishak Yildiz

**Affiliations:** 1 General Surgery, Istanbul Training and Research Hospital, Istanbul, TUR; 2 Gastroenterological Surgery, Basaksehir Cam and Sakura City Hospital, Istanbul, TUR; 3 General Surgery & Surgical Oncology, Basaksehir Cam and Sakura City Hospital, Istanbul, TUR

**Keywords:** severity, meld-na, meld-sodium, hepatopancreatobiliary, acute pancreatitis

## Abstract

Background and aim

Acute pancreatitis is a common inflammation of the pancreas which can be severe and even potentially mortal. High rates of mortality showed the importance of immediate identification of patients at high risk and led the clinicians to refer to various scoring systems. Our aim was to investigate a clinical predictive model using the Model for End-Stage Liver Disease-Sodium (MELD-sodium) scoring system, adapting it to acute pancreatitis patients referring to the systemic inflammatory nature of the disease and potential multi-organ failures in severe form.

Methods

Our multicenter study was designed retrospectively. The medical records were reviewed for the period of two years. Demographics, biochemical results, MELD-sodium scores and mortality rates were analysed.

Results

MELD-sodium score was found to be statistically correlated with both mortality and the severity of pancreatitis (p<0.001) and significant difference between both mild and severe (p<0.001), moderate and severe groups (p<0.001). Mortality was found to be significantly higher in patients with MELD-Na score when the cut-off value was accepted as ‘≥11’.

Conclusion

We found that MELD-sodium score was significantly associated with both severity of disease and mortality rates and also significantly effective between both mild/severe and moderate/severe groups which may be a guide for future multi-center reviews with larger patient and control groups, which can define the potential role of this non-invasive and easy-to-use predictive model in acute pancreatitis patients.

## Introduction

Acute pancreatitis (AP) is a common inflammation of the pancreas which can be severe and even potentially mortal. The severity of the disease usually depends on organ failures resulting from systemic response to the inflammation [1,2]. In recent years, high rates of mortality caused by AP showed the importance of immediate identification of patients at high risk. The prediction of the clinical course led the clinicians to refer to various scoring systems. Usually, 80% of the patients have a self-limiting disease and only need follow-up under hospitalization, nearly 20% of them have mortal complications [3], and death may be encountered in up to 50% of patients with severe AP [4,5]. According to recent literature, AP was responsible for 300,000 visits to the emergency department per year in the US [6] and 15000 cases in France [7], and generally 33.74 cases per 100,000 people in a year with a mortality of 1.60 [8] which increases the importance of early prediction of severity of the disease.

Revised Atlanta classification defined the clinical course of AP as mild, moderate and severe [9]. Mild form of the disease has an incidence of 70-75% with a low mortality rate because of the absence of major systemic complications. A more serious form of AP is encountered in 20-25% of the patients and is defined as a moderately-severe disease with high rates of morbidity and mortality [10] caused by organ failures. Severe AP refers to persistent (defined as more than 48 hours) organ failure, with a mortality rate of 50% [9,10]. Early identification of high-risk patients is important in foreseeing and preventing mortal complications. Existing predictive scoring systems usually depend on laboratory, clinical and radiological findings. Ranson, Imrie, Bedside Index of Severity of AP, the Balthazar score and the Glasgow score are some of them [3,11-13]. Laboratory findings such as C-reactive protein, proinflammatory cytokines and pancreatic enzymes have been found to be related with the severity of AP [14]. Unfortunately, none of the biochemical tests is consistent enough to show the accurate prediction of the severity of the disease. Even clinical risk factors such as obesity have been studied to result in more serious systemic response in the course of AP in animals [15]. But these scoring systems are complicated, cannot evaluate the patient immediately at the time of hospital admission and are not easy to use in the clinical practice of every institute.

The Model for End-Stage Liver Disease (MELD) score is a prospectively calculated scoring system which is derived from serum bilirubin, serum creatinine, and the international normalized ratio (INR) for prothrombin time. Generally, MELD scoring is used for predicting the severity of the hepatic failure and estimated risk of mortality, especially in patients involved in the liver transplant waiting list [16], replacing the CTP (Child-Turcotte-PUGH) score in deciding the priority of patients for liver donor [17]. The first incorporation of serum sodium in MELD scoring was resulted from a database study carried out in various transplant centers in the US among patients with end-stage liver disease for the better prediction of cirrhosis in patients with hepatocellular carcinoma, leading to a new scoring called ‘MELD-sodium’ [18]. In 2000, MELD-sodium was initially suggested as a prognostic feature in the outcome of patients who were candidates for transjugular intrahepatic portosystemic shunt [19]. The formulas for these scoring systems are as follows: MELD score = 3.78 × ln(serum bilirubin [mg/dL]) + 11.2 × ln(INR) + 9.57 × ln(serum creatinine [mg/dL]) + 6.43; MELD-Na score = MELD score + 1.59 (135 - Na) with maximum and minimum Na values of 135 and 120 mmol/L, respectively [20]. These formulas represent the advantage of MELD-sodium in predicting the clinical course in the targeted group of patients, which is the objective and case-specific variables without the need for an observer [21].

Objectives

Some of the published literature suggests that there is still no existing and universally accepted prognostic score which is reliable and convenient in a highly mortal disease such as AP [22]. In our study, our principal aim was to investigate a clinical predictive model using a well-known scoring system, adapting it to AP patients referring to the systemic inflammatory nature of the disease and potential multi-organ failures in severe form.

## Materials and methods

Following the approval of a local ethics committee, our multicenter study was designed retrospectively and performed in two different tertiary university hospitals. The medical records were reviewed for the period between 2019 and 2021. In this time period, acute pancreatitis had been diagnosed with clinical findings (abdominal pain, nausea, fever, etc.), laboratory tests (amylase, lipase, CRP, liver function tests and electrolytes) and contrasted computerized tomography (revealing pancreatic edema, necrosis or free fluid). Both biliary and nonbiliary-originated acute pancreatitis cases were involved in the study and demographics, biochemical results, MELD-sodium scores and mortality rates were analysed. Etiological origin (biliary vs nonbiliary) was studied and compared in means of its effect on MELD-sodium score. The scores were analysed between subgroups of AP patients in means of severity of the disease. The correlation between MELD-sodium scores and mortality rates was analysed and we also aimed to reach a possible cut-off value for the prediction of an increase in mortality rates, which may alert the clinician during follow-up. The specificity and sensitivity of our results were also compared with Ranson and BISAP scores of the same patients, which are well-known and commonly used scoring systems for AP.

The patients with former hepatopancreatobiliary surgery (laparoscopic/open cholecystectomy, choledochotomy, liver and pancreatic resections due to benign or malignant indication), diagnosed malignancy, malnutrition and history of recurrent hospital stays with chronic pancreatitis were excluded from the study group.

Statistical analysis

Statistical analysis of the data was performed using SPSS version 26.0 for Windows (IBM Corp., Armonk, NY, USA). The dispersion of variants was determined with the Kolmogorov-Smirnov test. The analysis between two independent groups with normal dispersion was performed with the Student's T-test and the analysis among more than three groups was performed with the One-Way ANOVA test. The analysis between two independent groups without normal dispersion was performed using the Mann-Whitney U test, while analysis was performed with the Kruskal-Wallis test among groups of more than three. When statistical significance was determined in end-group analyses, the Post Hoc Tukey test was used in groups with normal dispersion while the Mann-Whitney U test was performed among groups without normal dispersion for subgroup analyses. A two-sided p-value of < 0.05 was considered statistically significant. The Chi-Square test was used for the analysis of independent qualitative data. Correlation analysis of the data was performed using Spearman’s correlation test and considered as follows: “.00-.25: very weak, .25-.49: weak, .50-.69: moderate, .70-.89: strong, .90-1.0: very strong”.

## Results

A total of 248 patients who were diagnosed with acute pancreatitis were included in the study. Demographics of the patients are shown in Table 1.

**Table 1:** Demographics of the patients

	Min (Age)	Max (Age)	Median (Age)
Male (n: 142, 57%)	20	93	48
Female (n: 106, 43%)	32	78	62

Age or gender was not found to be significant in means of mortality or severity of pancreatitis.

Levels of serum amylase, lipase, C-reactive protein (CRP), urea, creatinine and calcium were analysed and not found to be statistically significant in means of mortality or severity of pancreatitis. The maximum-minimum and median levels including MELD-Na scores are shown in Table 2. One hundred and thirty of the patients were found to be of biliary origin (52.4%), while 118 were nonbiliary pancreatitis (47.6%). MELD-Na score was found to be statistically higher in the biliary group (Table 3).

**Table 2:** Laboratory data of the patients

	Min	Max	Median
C-reactive protein	0.4	446	45.0
Amylase	11	5656	329.0
Lipase	3	17100	492.0
Calcium	1.2	98	9.3
Urea	12	196	27.0
Creatinine	0.77	17	0.7

**Table 3:** Comparison of biliary and non-biliary origin MELD-Na: Model for End-Stage Liver Disease-Sodium

	Biliary (n: 130)	Non-biliary (n: 118)	P-value
MELD-Na score (median-IQR)	6 (8)	6 (0)	0.0000

Due to statistical analyses, MELD-Na score was found to be statistically correlated with both mortality and the severity of pancreatitis (p<0.001) (Tables 4, 5). The statistical analyses performed to reveal the origin of the significance among groups showed a significant difference between both mild and severe (p<0.001), moderate and severe (p<0.001) groups. Overall mortality was found to be 13 among 248 patients (5.2%).

**Table 4:** The relationship between MELD-Na score and mortality MELD-Na: Model for End-Stage Liver Disease-Sodium

	Mortality (-) (n: 235)	Mortality (+) (n: 13)	P-value
MELD-Na score (median- IQR)	6 (5)	21 (10)	<0.001

**Table 5:** The relationship between MELD-Na score and the severity of pancreatitis MELD-Na: Model for End-Stage Liver Disease-Sodium

	Mild (n: 158)	Moderate (n: 59)	Severe (n: 31)	P-Value
MELD-Na score (median-IQR)	6 (3)	6 (5)	21 (10)	<0.001

The correlation analysis revealed a weak positive correlation between the increase of MELD-Na score with mortality and the severity of pancreatitis (Table 6).

**Table 6:** The correlation between MELD-Na score and mortality and severity of disease MELD-Na: Model for End-Stage Liver Disease-Sodium

	Significance (p)	Correlation coefficient (R)
Mortality	<0.001	0.365
Severity	<0.001	0.484

Mortality was found to be significantly higher in patients with MELD-Na score when the cut-off value was accepted as ‘≥11’ (Table 7).

**Table 7:** The relationship between MELD-Na score and mortality due to cut-off value MELD-Na: Model for End-Stage Liver Disease-Sodium

	Mortality (-)	Mortality (+)	P-value
MELD-Na score (<11) (n,%)	200 (75.2%)	2 (14.3%)	<0.001
MELD-Na score (≥11) (n,%)	66 (24.8%)	12 (85.7%)	

**Figure 1:**
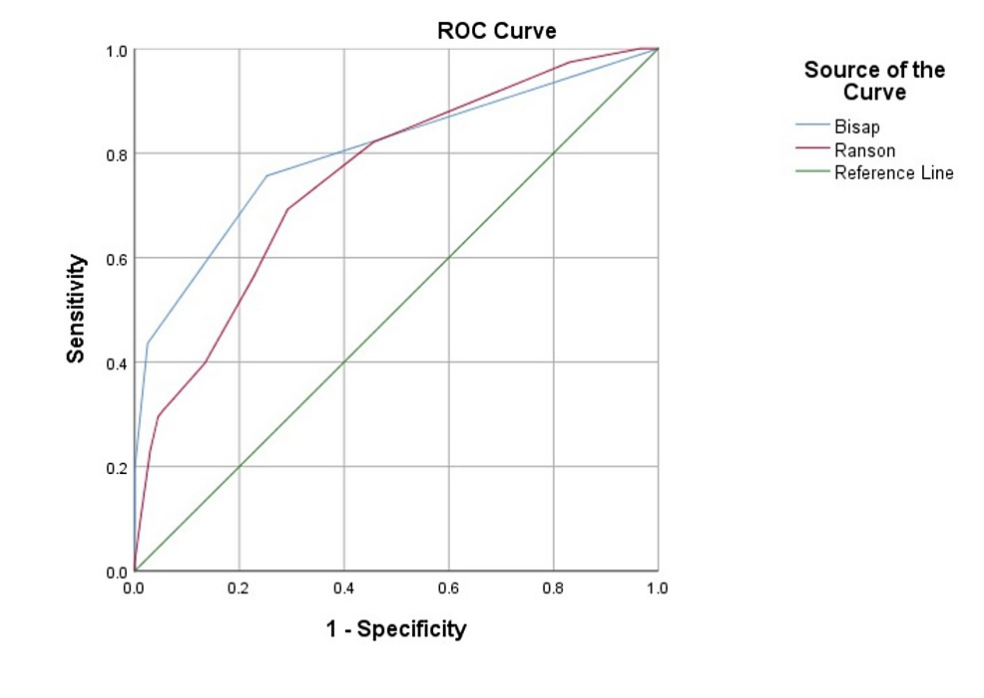
Comparison of MELD-Na score, ROC-Curve analysis BISAP: Bedside index of severity in acute pancreatitis MELD-Na: Model for End-stage Liver Disease-Sodium

MELD-Na scores were also compared with BISAP and Ranson scores. ROC-Curve analysis showed sensitivity and specificity as 75% and 74%, respectively when cut-off MELD-Na score was accepted as ‘11’ and BISAP as ‘1’. Sensitivity and specificity were found to be 69% and 71%, respectively when the average Ranson score was accepted as ‘4’ (Figure 1).

## Discussion

Pancreas can be considered as the most important digestive organ of the gastrointestinal system. Major digestive capability of the organ depends on its endocrine and exocrine functions. Unfortunately, in case of an anatomic, functional or systemic disorder, these highly effective enzymes may also cause damage to the pancreatic tissues [5,23]. According to the revised Atlanta classification concerning the severity of AP, the clinical process is classified mainly into three groups such as mild, moderate and severe [9]. In mild and moderate forms of AP, autoenzymes affect pancreatic tissues and adjacent organs which may be considered as a local disease, while the severe form of AP is characterized by multiple organ failures due to systemic effects in course of the disease [4,5]. Early diagnosis and initial treatment are known to have great importance in decreasing the rates of morbidity and mortality [24]. Therefore, various clinical, radiological and laboratory parameters have been studied to predict the course of the disease as early as possible.

There are numerous scoring systems such as Ranson, APACHE II, Glasgow-Imrie, CTSI, HAPS, BISAP, etc. usually preferred according to the practice of the centers and clinicians. But one of the major limitations of these systems is the fact that all of them have been suggested before the development of revised Atlanta classification which defines AP as mild, moderately severe and severe, unlike the original Atlanta classification which had defined AP only as mild and severe [1]. For example, in different studies, both Lautz et al. and Khanna et al. showed the inefficiency of existing systems in predicting the severity of AP, especially in pediatric patients groups in whom the incidence is rare but may end in high rates of morbidity and mortality [25,26]. These limitations led the clinicians to decide that existing scoring systems are not effective anymore in predicting the severity of AP and look for new perspectives [22]. Although we did not find any significance in our retrospective analysis in laboratory findings, Ammori et al. showed the importance of serum calcium levels, relating low levels with high complication rates [27]. Hong et al. suggested their new AP severity scoring using serum albumin, blood urea nitrogen, pleural effusion and systemic inflammatory response syndrome, with a higher rate of prediction when compared to APACHE II, Glasgow and BISAP [28]. The difficulties in means of a relatively subjective approach in existing scoring systems, directed the researchers to work on machine learning systems, such as Pearce et al. who used kernel logistic regression analysis to predict the severity of AP, achieving a higher score than APACHE II [29]. These high rates of clinical consistency in the course of the disease led the researchers like Kui et al. to apply even artificial intelligence in predicting the severity of AP [1].

The Model for End-Stage Liver Disease (MELD) score was mainly developed and used for the risk estimation of patients who are in the liver transplant waiting list [16]. Later, serum level of sodium was added to MELD in 2000 for a better clinical prediction in patients with liver failure [18,19]. MELD-sodium score involves the parameters of serum bilirubin, serum creatinine, the international normalized ratio for prothrombin time (INR) and sodium. Although it depends mainly on hepatobiliary system with similar parameters, MELD-Na has not been researched in patients with AP. In a recent study in 2021, in guidelines for the management of patients with acute pancreatitis, Jaber et al. showed that increased bilirubin levels may be associated with multiorgan failure in cancer patients with ascites [7]. The fact that severe AP is a systemic hepatopancreatobiliary disease leading to multiorgan failure, we decided to search the predictive value of MELD-sodium in the severity of AP.

In our study, which we think as the first research studying the potential predictive value of MELD-Na in AP severity, we found that MELD-Na score was significantly associated with both severity of disease and mortality rates. MELD-Na was also found to be significantly effective in both mild/severe and moderate/severe groups. Although it was weak, our analysis revealed a correlation between the increase of MELD-Na score and mortality and the severity of pancreatitis. In our statistical analysis, the significant cut-off value of MELD-Na score was found to be ‘11’ in predicting the risk of mortality. We also compared MELD-Na score with well-known scoring systems such as BISAP and Ranson and showed that MELD-Na was more similar to BISAP when compared to Ranson in our study group.

The limitations of our study are the retrospective design, limited patient number of study group and the existing limits of MELD-Na itself in its original role in clinical practice. The strengths of our study are its multi-center design and the role of a well-known and objective scoring system which has been used safely in tertiary centers worldwide for years.

## Conclusions

Our statistically significant results may be a guide for future multi-center reviews with larger patient and control groups, which can define the potential role of this non-invasive and easy-to-use predictive model in acute pancreatitis patients. In conclusion, the MELD-Na score can be one of the predictors of severity and survival in AP and can be used as an evaluation tool at the time of hospital admission which may be an advantage to other scoring systems that need a few hours time following diagnosis.
